# Supplementary material for: Investigating the Design of a Photoplethysmography Device for Vital Sign Monitoring
Source: Sensors (Basel). 2025 Mar 18;25(6):1875. doi: 10.3390/s25061875 (PMC11946623; doi:10.3390/s25061875)
Supplement: Supplementary file 1 [file sensors-25-01875-s001.zip › sensors-3481212-supplementary.pdf]

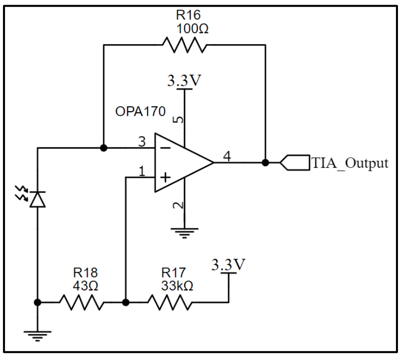

Figure S1: Transimpedance Amplifier Design.

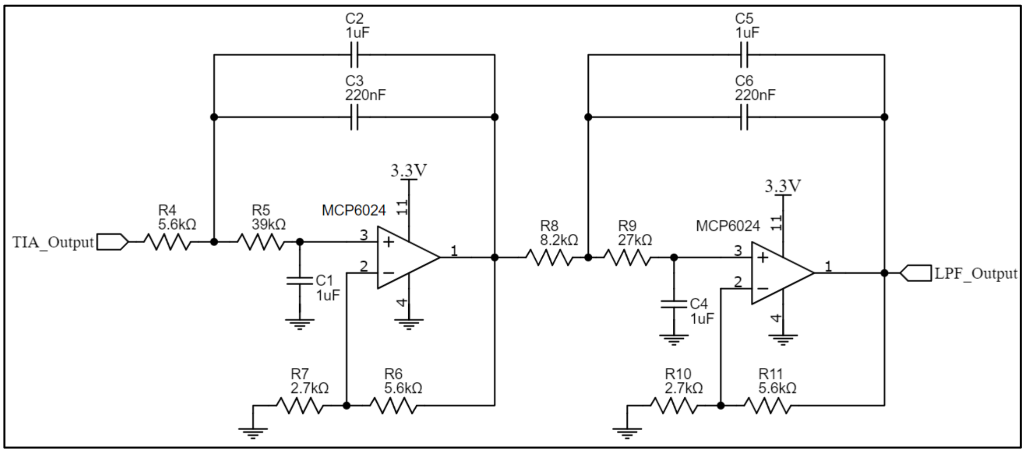

Figure S2: Low-Pass Filter Design.

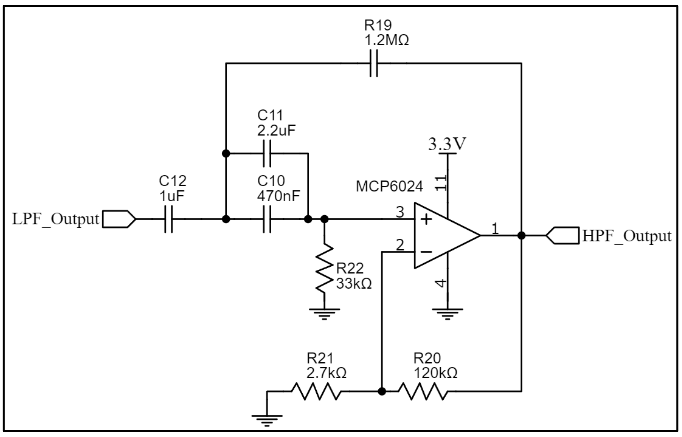

Figure S3: High-Pass Filter Design.

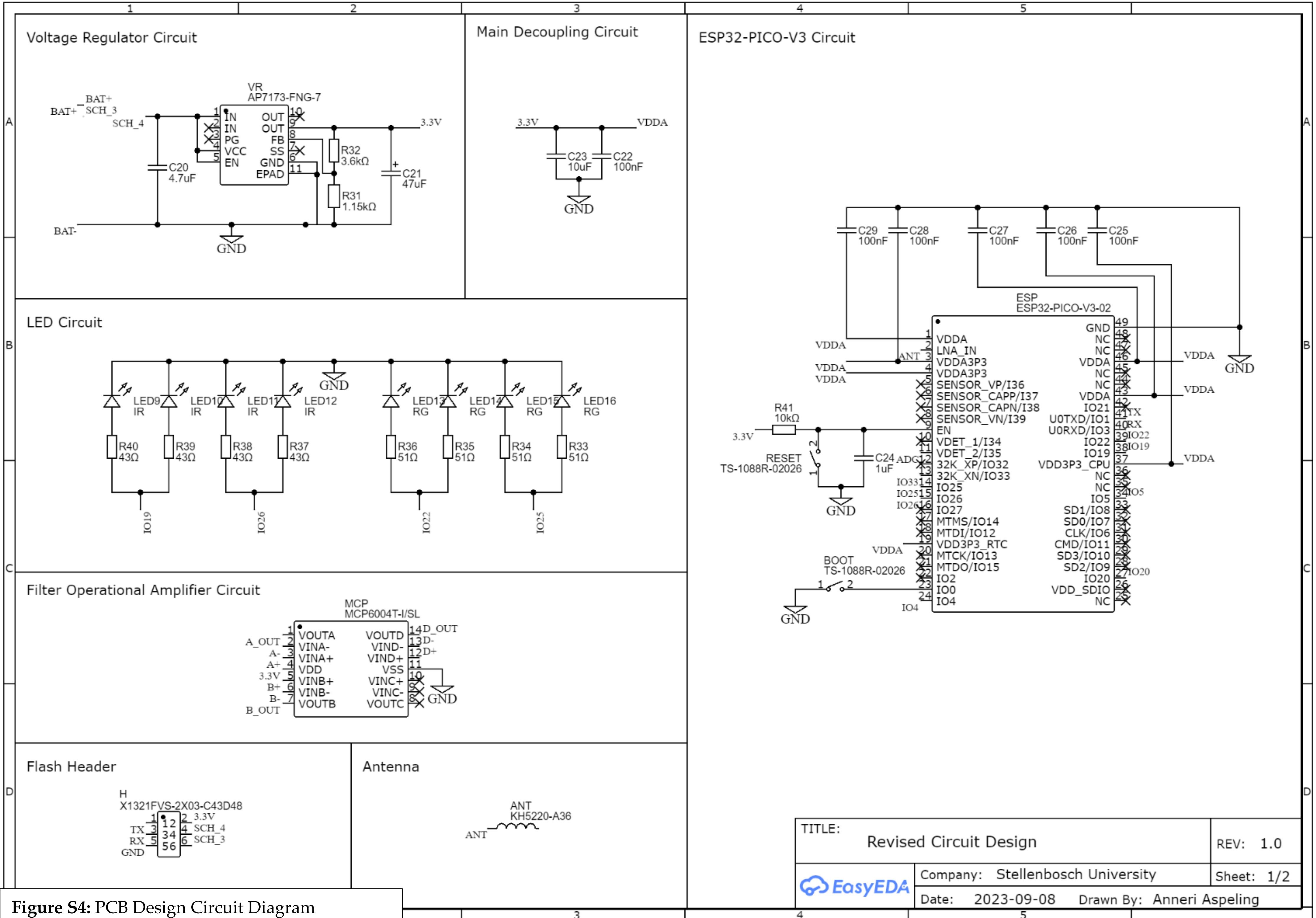

Figure S4: PCB Design Circuit Diagram

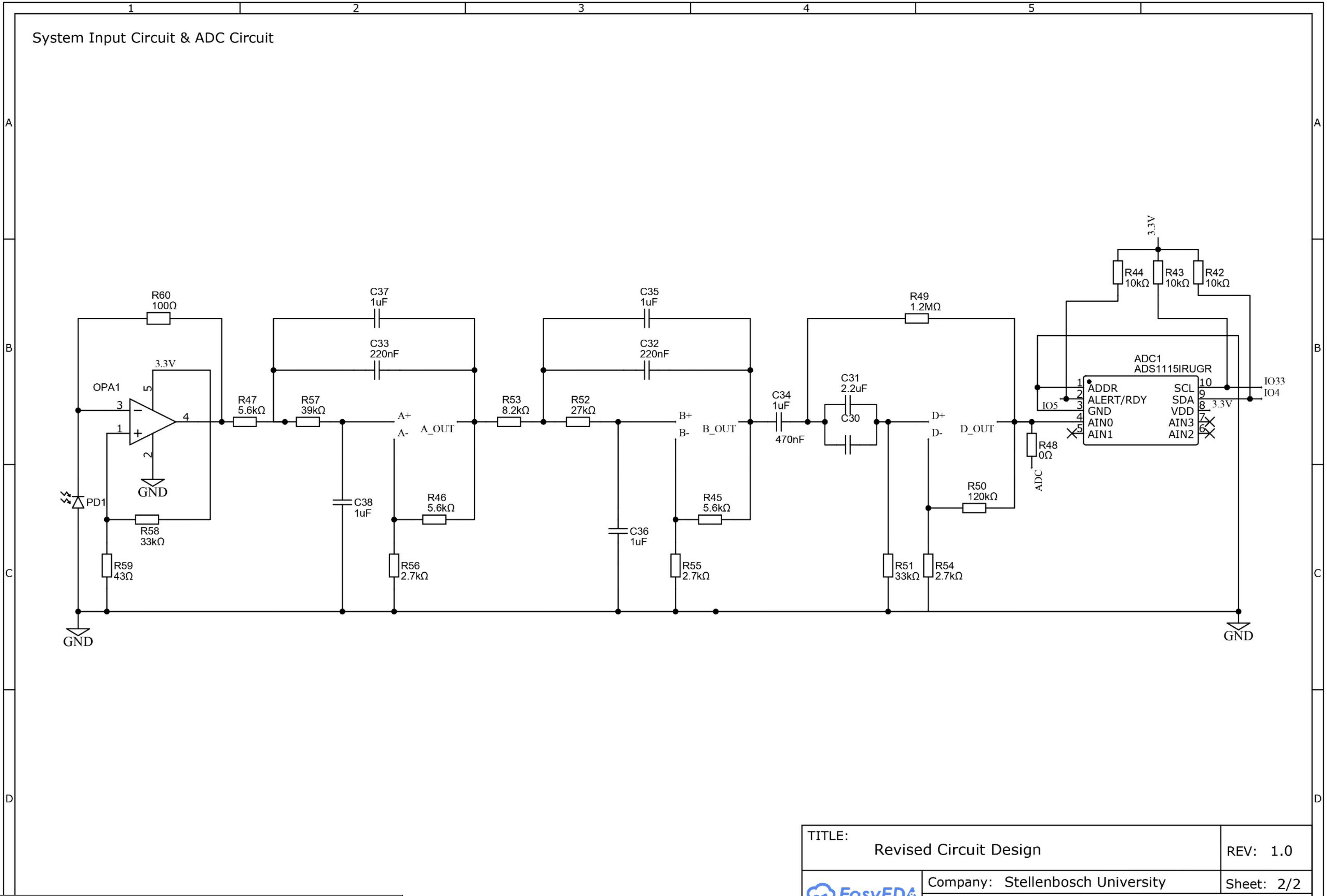

Figure S4: PCB Design Circuit Diagram

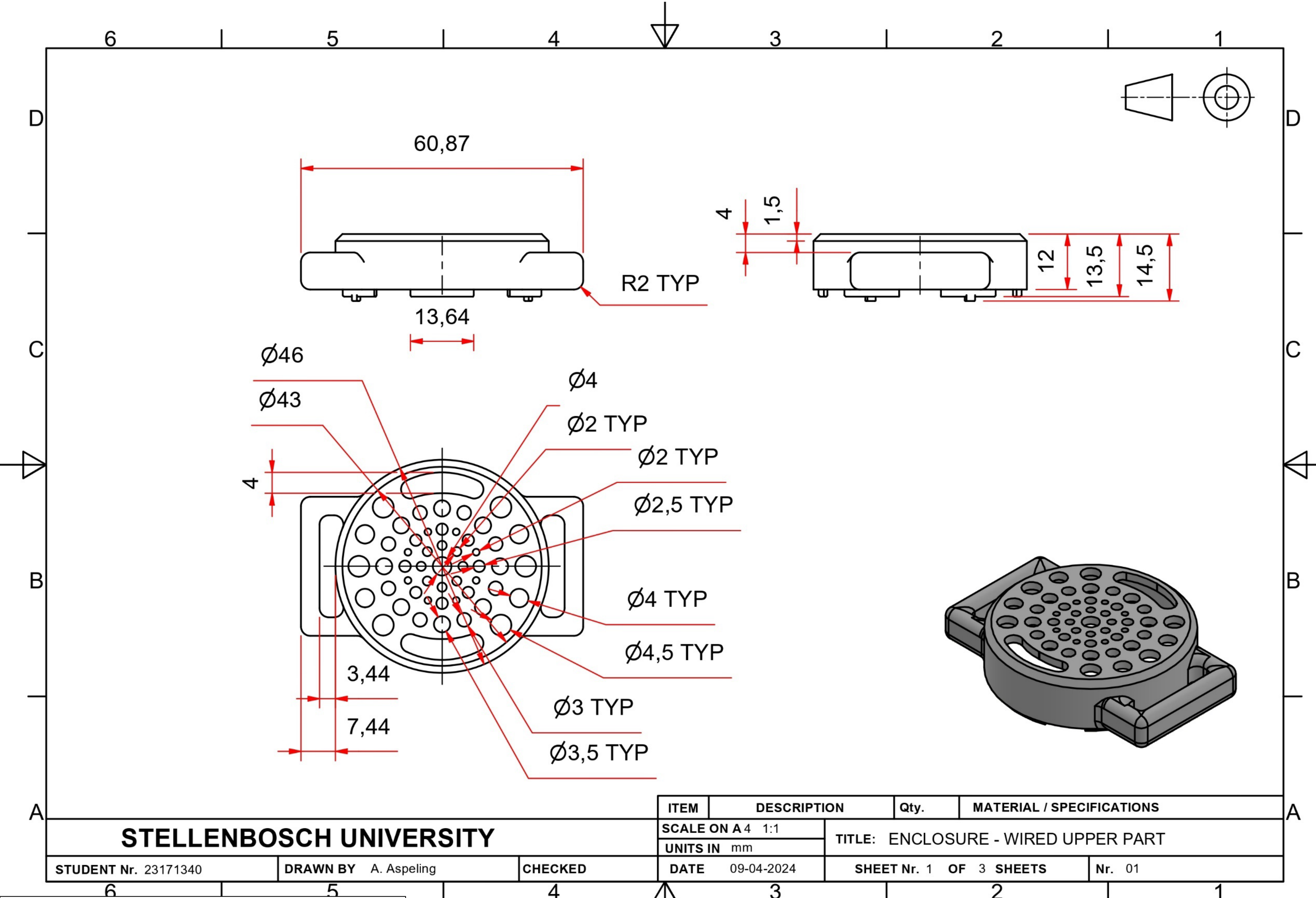

Figure S5: Enclosure Technical Drawings

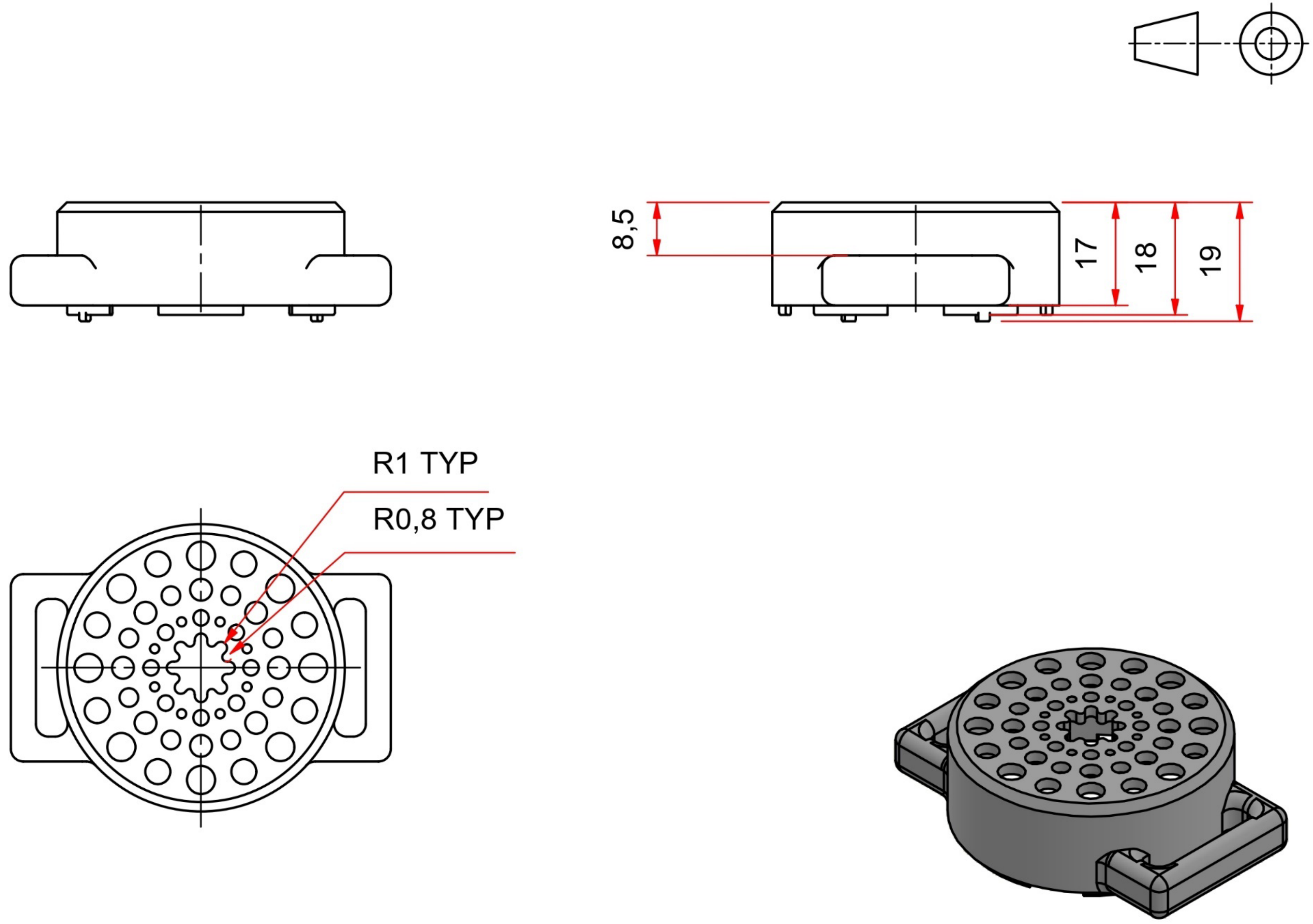

NOTE: ONLY THE DIMENSIONS THAT ARE DIFFERENT FROM
SHEET 1 ARE ANNOTATED

ITEM	DESCRIPTION	Qty.	MATERIAL / SPECIFICATIONS

**STELLENBOSCH UNIVERSITY**		**SCALE ON A4** 1:1 / **UNITS IN** mm	**TITLE:** ENCLOSURE - WIRELESS UPPER PART
**STUDENT Nr.** 23171340	**DRAWN BY** A. Aspeling	**CHECKED**	**DATE** 09-04-2024
**SHEET Nr.** 2 **OF** 3 **SHEETS**	**Nr.** 02		

Figure S5: Enclosure Technical Drawings

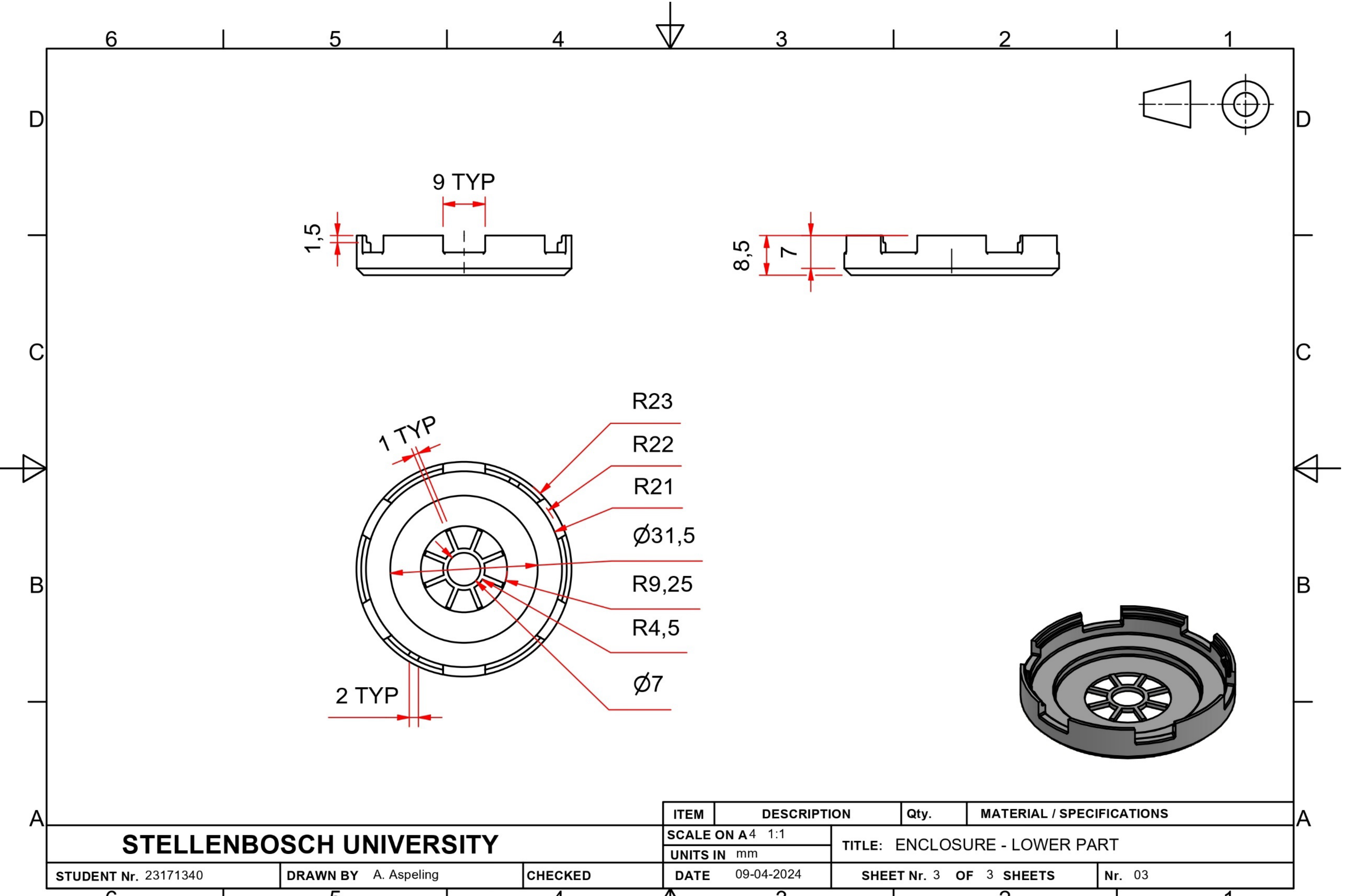

Figure S5: Enclosure Technical Drawings

Average Preliminary Performance Evaluation Results

Table S1 - Average qualitative results for the preliminary performance evaluation.

Sensor-to-Skin Contact Pressure	Body Location	Photodetector Lens Shape	Visible Light Source Colour	Light Source Arrangement	Light Source Brightness	Results			
						FFT Ranking (RG)	FFT Ranking (IR)	Time Signal Ranking (RG)	Time Signal Ranking (IR)
1	Finger	Flat	Green	2	100	1.83	2.67	4.17	3.33
					175	2.00	1.67	4.00	2.83
					250	2.33	2.83	3.83	2.67
				4	100	1.83	2.33	3.67	3.50
					175	2.00	1.50	3.67	3.00
					250	2.33	2.67	3.50	3.83
		Dome	Green	2	100	2.50	2.17	4.83	4.67
					175	3.00	2.33	4.33	4.33
					250	3.00	2.50	4.50	3.83
				4	100	3.00	2.83	4.50	3.83
					175	3.00	3.33	4.67	3.50
					250	2.50	2.00	4.83	3.17
	Wrist	Flat	Red	2	100	2.17	2.17	5.00	4.83
					175	2.83	2.17	5.00	4.83
					250	1.67	3.67	4.67	4.83
				4	100	2.83	3.17	4.67	4.83
					175	1.67	2.17	4.50	4.67
					250	2.50	2.83	4.67	4.67

Sensor-to-Skin Contact Pressure	Body Location	Photodetector Lens Shape	Visible Light Source Colour	Light Source Arrangement	Light Source Brightness	Results			
						FFT Ranking (RG)	FFT Ranking (IR)	Time Signal Ranking (RG)	Time Signal Ranking (IR)
		Dome	Red	2	100	2.17	3.17	4.17	4.83
		Dome	Red	2	175	3.67	2.50	4.67	4.67
		Dome	Red	2	250	3.33	3.67	5.00	4.50
		Dome	Red	4	100	2.50	2.50	4.67	4.67
		Dome	Red	4	175	2.83	2.17	4.50	4.83
		Dome	Red	4	250	2.50	2.17	4.83	4.67
2	Finger	Flat	Green	2	100	1.83	1.17	4.00	2.50
2	Finger	Flat	Green	2	175	2.17	1.17	3.50	1.67
2	Finger	Flat	Green	2	250	1.67	1.17	2.67	2.17
2	Finger	Flat	Green	4	100	1.17	2.83	2.67	2.50
2	Finger	Flat	Green	4	175	2.00	1.33	2.33	2.67
2	Finger	Flat	Green	4	250	2.00	2.50	2.67	3.50
2	Finger	Dome	Green	2	100	2.00	1.67	4.33	4.33
2	Finger	Dome	Green	2	175	2.83	1.83	4.33	3.33
2	Finger	Dome	Green	2	250	3.67	1.50	4.67	3.00
2	Finger	Dome	Green	4	100	2.33	1.33	4.50	3.33
2	Finger	Dome	Green	4	175	3.83	1.33	4.67	2.67
2	Finger	Dome	Green	4	250	2.33	1.33	4.67	2.33
2	Wrist	Flat	Red	2	100	3.00	2.67	4.33	4.67
2	Wrist	Flat	Red	2	175	3.17	2.50	4.67	4.83
2	Wrist	Flat	Red	2	250	3.50	2.67	4.67	4.67

Sensor-to-Skin Contact Pressure	Body Location	Photodetector Lens Shape	Visible Light Source Colour	Light Source Arrangement	Light Source Brightness	Results			
						FFT Ranking (RG)	FFT Ranking (IR)	Time Signal Ranking (RG)	Time Signal Ranking (IR)
				4	100	3.00	3.33	4.67	5.00
					175	3.00	3.17	4.67	4.83
					250	3.67	3.83	5.00	4.33
		Dome	Red	2	100	3.33	3.50	4.33	4.67
					175	4.00	3.50	4.50	4.67
					250	3.33	3.67	4.67	4.83
				4	100	3.00	2.00	4.50	4.83
					175	3.33	1.83	4.67	4.83
					250	2.67	3.83	4.83	4.83
3	Finger	Flat	Green	2	100	2.00	1.83	3.33	2.00
					175	1.33	2.00	2.33	1.50
					250	1.50	1.17	2.17	1.67
				4	100	1.83	1.50	2.50	2.83
					175	1.83	1.83	2.17	1.67
					250	1.83	2.67	2.33	3.33
		Dome	Green	2	100	3.33	2.33	4.67	3.67
					175	2.00	1.50	4.50	3.00
					250	3.50	1.50	4.33	2.67
				4	100	2.83	2.17	4.33	3.17
					175	3.00	1.50	4.83	2.33
					250	2.17	1.33	4.33	2.17

Sensor-to-Skin Contact Pressure	Body Location	Photodetector Lens Shape	Visible Light Source Colour	Light Source Arrangement	Light Source Brightness	Results			
						FFT Ranking (RG)	FFT Ranking (IR)	Time Signal Ranking (RG)	Time Signal Ranking (IR)
	Wrist	Flat	Red	2	100	2.50	2.67	4.50	4.67
					175	3.17	3.17	4.67	4.67
					250	2.67	3.50	4.50	4.67
				4	100	3.00	2.83	4.67	4.83
					175	4.17	2.17	4.67	4.83
					250	3.00	2.50	4.83	4.83
		Dome	Red	2	100	3.00	3.50	4.33	4.67
					175	2.50	2.83	4.67	4.50
					250	3.83	4.17	4.50	4.50
				4	100	2.83	3.00	4.50	5.00
					175	3.33	2.50	4.67	4.50
					250	3.83	2.83	4.67	4.83
1	Finger	Flat	Red	2	100	2.67	1.67	4.33	3.50
					175	2.33	2.33	4.17	3.17
					250	2.17	1.83	3.50	3.67
				4	100	2.17	2.50	4.17	4.17
					175	1.83	2.17	3.67	4.17
					250	2.00	2.00	3.83	4.33
		Dome	Red	2	100	2.50	3.00	4.67	4.50
					175	2.17	1.83	4.50	4.33
					250	2.17	2.33	5.00	4.33

Sensor-to-Skin Contact Pressure	Body Location	Photodetector Lens Shape	Visible Light Source Colour	Light Source Arrangement	Light Source Brightness	Results			
						FFT Ranking (RG)	FFT Ranking (IR)	Time Signal Ranking (RG)	Time Signal Ranking (IR)
				4	100	2.50	2.00	4.50	4.67
					175	2.33	2.33	4.67	4.50
					250	2.00	2.67	4.83	4.50
	Wrist	Flat	Green	2	100	2.83	2.50	4.33	4.67
					175	3.00	2.83	4.33	4.17
					250	1.67	3.50	4.50	4.67
				4	100	3.17	3.33	4.50	4.33
					175	2.50	2.50	4.83	4.67
					250	2.50	3.83	4.17	4.83
		Dome	Green	2	100	3.17	2.33	4.67	4.67
					175	3.50	3.33	4.67	4.83
					250	3.33	2.17	4.50	4.50
				4	100	2.50	2.00	4.83	4.50
					175	2.83	3.33	4.67	4.83
					250	2.67	2.50	4.67	4.83
2	Finger	Flat	Red	2	100	1.67	1.50	3.17	2.50
					175	1.50	1.83	3.17	2.50
					250	1.67	1.67	3.00	2.17
				4	100	1.33	1.50	3.00	3.00
					175	2.67	1.67	3.50	3.33
					250	3.00	2.33	3.50	3.50

Sensor-to-Skin Contact Pressure	Body Location	Photodetector Lens Shape	Visible Light Source Colour	Light Source Arrangement	Light Source Brightness	Results			
						FFT Ranking (RG)	FFT Ranking (IR)	Time Signal Ranking (RG)	Time Signal Ranking (IR)
		Dome	Red	2	100	3.00	1.67	4.17	4.17
					175	2.67	2.33	4.33	4.00
					250	3.00	2.17	4.50	3.33
				4	100	2.67	1.50	4.67	3.33
					175	2.00	2.67	4.33	3.83
					250	2.33	2.50	4.00	4.00
	Wrist	Flat	Green	2	100	2.17	3.00	4.17	5.00
					175	2.33	2.00	4.33	4.50
					250	3.00	2.83	4.67	4.33
				4	100	2.50	3.17	4.50	4.83
					175	3.17	2.17	4.33	4.50
					250	2.50	2.67	4.67	4.83
		Dome	Green	2	100	2.17	2.83	4.83	4.67
					175	2.33	2.17	4.83	4.83
					250	2.50	3.67	4.67	4.50
				4	100	2.00	2.00	4.50	5.00
					175	2.83	3.17	4.67	4.50
					250	2.67	2.83	4.83	4.83
3	Finger	Flat	Red	2	100	1.33	1.67	3.17	2.17
					175	1.67	1.33	2.83	1.83
					250	1.83	1.50	2.33	2.17

Sensor-to-Skin Contact Pressure	Body Location	Photodetector Lens Shape	Visible Light Source Colour	Light Source Arrangement	Light Source Brightness	Results			
						FFT Ranking (RG)	FFT Ranking (IR)	Time Signal Ranking (RG)	Time Signal Ranking (IR)
				4	100	1.17	1.67	2.67	2.33
					175	1.67	2.00	2.17	2.83
					250	1.67	1.83	2.83	3.67
		Dome	Red	2	100	2.50	1.50	4.67	3.83
					175	2.83	1.50	4.33	3.67
					250	1.83	1.50	3.83	3.17
				4	100	1.67	1.83	4.33	3.83
					175	1.83	2.00	3.83	3.00
					250	1.83	2.17	3.67	3.67
	Wrist	Flat	Green	2	100	2.17	3.83	4.50	4.50
					175	2.67	3.00	4.67	4.83
					250	3.33	2.33	4.83	4.83
				4	100	2.33	2.67	4.67	4.83
					175	3.50	2.67	4.33	4.83
					250	2.50	3.00	4.67	5.00
		Dome	Green	2	100	3.17	2.50	4.17	4.67
					175	3.17	2.83	4.17	4.50
					250	2.33	3.50	4.50	4.83
				4	100	3.33	2.50	4.83	4.67
					175	2.33	3.00	4.67	5.00
					250	3.17	2.67	4.50	4.83
